# Assessing the Effectiveness of Chemical Marker Extraction from Amazonian Plant Cupuassu (*Theobroma grandiflorum*) by PSI-HRMS/MS and LC-HRMS/MS

**DOI:** 10.3390/metabo13030367

**Published:** 2023-03-01

**Authors:** Nerilson M. Lima, Gesiane S. Lima, Gabriel F. dos Santos, Gagan Preet, Lanaia I. L. Maciel, Teresinha de Jesus A. S. Andrade, Marcel Jaspars, Andrea R. Chaves, Boniek G. Vaz

**Affiliations:** 1 Institute of Chemistry, Federal University of Goias, Goiânia 74690-900, Brazil; 2 Marine Biodiscovery Centre, Department of Chemistry, University of Aberdeen, Aberdeen AB24 3UE, UK; 3 Federal Institute of Maranhão, Timon Campus, Timon 65635-468, Brazil

**Keywords:** ambient mass spectrometry ionization, cupuassu, *Theobroma grandiflorum*, untargeted metabolomics, paper spray ionization mass spectrometry, liquid chromatography–mass spectrometry

## Abstract

Employing a combination of liquid chromatography electrospray ionization and paper spray ionization high-resolution tandem mass spectrometry, extracts from cupuassu (*Theobroma grandiflorum*) pulp prepared with either water, methanol, acetonitrile or combinations thereof were subjected to metabolite fingerprinting. Among the tested extractors, 100% methanol extracted preferentially phenols and cinnamic acids derivatives, whereas acetonitrile and acetonitrile/methanol were more effective in extracting terpenoids and flavonoids, respectively. And while liquid chromatography- mass spectrometry detected twice as many metabolites as paper spray ionization tandem mass spectrometry, the latter proved its potential as a screening technique. Comprehensive structural annotation showed a high production of terpenes, mainly oleanane triterpene derivatives. of the mass spectra Further, five major metabolites with known antioxidant activity, namely catechin, citric acid, epigallocatechin-3′-glucuronide, 5,7,8-trihydroxyflavanone, and asiatic acid, were subjected to molecular docking analysis using the antioxidative enzyme peroxiredoxin 5 (PRDX5) as a model receptor. Based on its excellent docking score, a pharmacophore model of 5,7,8-trihydroxyflavanone was generated, which may help the design of new antioxidants.

## 1. Introduction

Cupuassu (*Theobroma grandiflorum* Willd. ex Spreng. K.), a member of the Malvaceae family, is a tropical fruit widely grown in the Amazon Rainforest, especially in northern Brazil, and is commonly related to cacao (*Theobroma cacao* L.). *T. grandiflorum* has brown-colored fruit with a length of around 12–25 cm, and a weight of 1 to 2 kg per fruit. The pulp presents a yellowish-white color and is highly appreciated due to its excellent texture and intense flavor [1,2]. Cupuassu is of great economic importance, since the pulp is of great interest in the cosmetics industry in the preparation of fragrances and widely used in food technology due to its nutritional value and commercial applications in the preparation of juices, sorbets, ice creams, jams, liquors, sweets, and derived products [3,4,5]. The fruit pulp has a characteristic acidic taste and is a rich source of antioxidant polyphenols such as catechin, 8-hydroxy-flavones, proanthocyanidins, flavan-3-ols, and citric acid [5,6,7].

Considering the biotechnological potential of cupuassu and the scarcity of chromatographic and spectrometric data that allow for a comprehensive metabolic characterization of its fruits, there is a need to develop analytical methodologies and apply state-of-the-art technologies for the qualitative and quantitative determination of metabolites for this widely consumed fruit. Mass spectrometry (MS)-based analytical platforms stand out for their ability to dereplicate complex samples and detect trace-level molecules due to their high selectivity, sensitivity, speed, and versatility [8,9,10,11]. In addition to its high resolving power and the ability to provide high-precision molecular formulas, this technique can be hyphenated to chromatographic separation equipment, increasing its metabolic coverage capacity [10,11,12].

Aiming to reduce the multistep, laborious, and high-solvent-consumption process usually involved in conventional MS methods, ambient mass spectrometry (AMS) techniques have been increasingly applied to the analysis of natural products [13,14,15]. Paper spray ionization (PSI) is one of the most used ambient ionization techniques in food chemistry and metabolite fingerprinting of fruits, as it provides reliable and rapid results at low cost [16,17,18,19,20,21,22].

For PSI-MS analysis, a relatively small sample volume (less than about 50 μL) is placed onto a triangular piece of paper held in front of the mass spectrometer inlet. Sample ionization is conducted by applying a high voltage to the paper moistened with a small amount of solution. There are different ways to load the sample: it can be directly loaded onto the paper surface, combined with the wetting solution, or removed from surfaces using the paper as a wipe [21,23].

The aim of this study was to apply liquid chromatography–high-resolution mass spectrometry (LC-HRMS/MS) and paper spray ionization–high-resolution mass spectrometry (PSI-HRMS/MS) to the metabolic characterization of cupuassu (*T. grandiflorum*) pulp extracted with different solvents. Mass spectra obtained were comprehensively using various dereplication tools. Finally, the holistic biological properties of the compounds identified were predicted by applying molecular docking approaches.

## 2. Materials and Methods

### 2.1. Chemicals

HPLC-grade methanol (MeOH) and acetonitrile (ACN) were acquired from Tedia (Fairfield, CT, USA). Ultrapure water was obtained using a water purification system (Master System MS2000, Gehaka, São Paulo, Brazil).

### 2.2. Sample Preparation

*Theobroma grandiflorum* fruits were purchased from a local market in Manaus, Brazil. The pulp of *T. grandiflorum* fruits was sliced manually using a sterile knife and dried at 30 °C for 48 h. Aliquots of 1 g of dried pulp were extracted separately using 10 mL of water (H_2_O), methanol (MeOH), acetonitrile (ACN), H_2_O/MeOH (1:1, *v*/*v*), MeOH/ACN (1:1, *v*/*v*), ACN/H_2_O (1:1, *v*/*v*), and H_2_O/MeOH/ACN (1:1:1, *v*/*v*/*v*) [24,25]. The extracts were diluted to a concentration of 500 µg mL^−1^ prior to LC-HRMS/MS and PSI-HR-MS/MS analyses.

### 2.3. LC-MS Analysis

LC-MS/MS analyses were performed as described in a previous study [26] on an HPLC-UV 1220 Infinity II instrument (Agilent Technologies, Santa Clara, CA, USA) coupled with a Q-Exactive hybrid quadrupole-Orbitrap high-resolution mass spectrometer (Thermo Scientific, Waltham, MA, USA) using an electrospray ionization source. An InfinityLab Poroshell 120 EC-C18 column (4.6 × 100 mm × 2.7 μm Agilent) was used in this study. All samples were analyzed using a gradient elution program. The binary mobile phase comprised A (water with 0.1% formic acid) and B (methanol). The mobile phase was acidified (0.1% formic acid) in order to reduce the issue of coelution and tailing effect. The gradient elution started at 5% B and was linearly increased to 100% B in 40 min and kept constant for 10 min at 100% B. The eluent was then restored to the initial conditions within 10 min. The flow rate was set at 0.3 mL min^−1^. The injection volume was 30 μL, and the column temperature was set at 35 °C. The ESI source conditions were set as follows: spray voltage, 3.5 kV (in both ionization modes); capillary temperature, 250 °C (positive mode) and 320 °C (negative mode); S-lens RF level, 60 V (in both ionization modes); sheath gas flow rate, 47 L min^−1^ (positive mode) and 35 L min^−1^ (negative mode); and aux gas flow rate, 11 L min^−1^ (positive mode) and 10 L min^−1^ (negative mode). In both ESI positive and negative modes, high-resolution mass spectra were obtained in the full MS/data-dependent-MS^2^ (dd-MS^2^) mode. The mass range in the full MS scanning experiments was *m*/*z* 100–1200. The top 5 (TopN, 5; loop count, 5) most abundant precursors were sequentially transferred for collision-induced fragmentation acquisition. The collision energies for target analytes were 20, 30, and 35 eV. Resolving power was set at 140,000 and 70,000 for full MS and dd-MS^2^ acquisitions, respectively.

### 2.4. PSI-MS

PSI-MS analysis was performed using previously described protocols [27,28,29]. The membrane was cut into an equilateral triangle with 1 cm sides and held by a metal clip connected to the voltage source of the mass spectrometer. The membrane was positioned approximately 4 mm from the mass spectrometer inlet. The extract (15 μL) was deposited on the membrane and dried under ambient conditions for 1 min. Subsequently, the voltage source was turned on, and 15 μL of acidified methanol (0.1% formic acid, *v*/*v*) was used as a spray solvent. The analyses were performed in triplicate using a Q-Exactive hybrid quadrupole-Orbitrap mass spectrometer (Thermo Scientific) with the following instrumental parameters: positive ionization mode; spray voltage, 3.5 kV; acquisition time, 1 min; capillary temperature, 275 °C; capillary voltage, 38 V; and tube lens, 50 V.

### 2.5. Compound Characterization

The files acquired by the Q-Exactive hybrid quadrupole-Orbitrap mass spectrometer for the cupuassu extracts were converted from raw to mzML format using MSConvert software (ProteoWizard, Palo Alto, CA, USA). Then, the data were processed using MZmine software, version 2.53. We used metadata to organize compound information according to the online workflow available in the GNPS (Global Natural Products Social Molecular Networking) documentation. Structural annotation and data mining were performed using the experimental and in silico tools available in the GNPS platform [26].

### 2.6. Molecular Docking

Molecular docking analysis was performed using Autodock Vina v.1.2.0 (The Scripps Research Institute, La Jolla, CA, USA) docking software [30]. The receptor site was predicted using LigandScout 4.4 (Inte: Ligand) advanced software [31], which identifies putative binding pockets by creating a grid surface and calculating the buriedness value of each grid point on the surface.

The resulting pocket grid consists of several clusters of grid points rendered using an iso surface connecting the grid points to each other. The iso surface represents an empty space that may be suitable for creating a pocket. The x-ray crystal structures of tyrosinase from *Bacillus megatarium* (PDB: 3NM8) [32] and human peroxiredoxin 5, a novel type of mammalian peroxiredoxin (PDB: 1HD2) [33], were retrieved from the Protein Data Bank and utilized to perform docking simulations.

The box center and size coordinates for PDB: 3NM8 were −5.59208 Å × −3.33416 Å × 10.2111 Å and 31.5668 Å × 22.6662 Å × 23.0593 Å, and those for PDB: 1HD2 were 18.9015 Å × 44.192 Å × 28.1802 Å and 12.81 Å × 17.1255 Å × 15.8749 Å, respectively, around the active site. The following default search parameters were used: 10 binding modes; exhaustiveness, 8; and maximum energy difference, 3 kcal/mol. Chimera 1.16 and UCSF_USA [34,35] were used for visualization and calculation of protein-ligand interactions.

### 2.7. 3D Pharmacophore Model Generation

LigandScout 4.4 by Inte Ligand advanced software (Wolber and Langer, Vienna, Austria) [31] was used to generate a 3D pharmacophore model. The espresso algorithm was used to generate a ligand-based pharmacophore. The compatibility of the generated pharmacophore model with the pharmacophore hypothesis was determined using default settings for LigandScout. The relative pharmacophore-fit scoring function, and merged feature pharmacophore type were used for ligand-based pharmacophore creation, and the feature tolerance scale factor was set to 1.0. The best model was selected from the 10 generated models.

## 3. Results and Discussion

### 3.1. Comprehensive Metabolite Annotation of Cupuassu (T. grandiflorum) Pulp Extract

Paper spray ionization–high-resolution mass spectrometry (PSI-HRMS/MS) liquid chromatography–high-resolution mass spectrometry (LC-HRMS/MS) data were used for the comprehensive structural annotation of cupuassu pulp extract. The metabolite annotation was based on accurate mass (*m*/*z*) and MS/MS fragmentation pattern and compared with data from the literature. UV spectra and chromatographic retention time data obtained from the chromatographic analysis were used to distinguish isobaric and isomeric compounds and improve the confidence level of the metabolic annotations [36,37,38,39]. Tandem mass spectrometry (MS/MS) data collected in positive ionization mode (ESI(+)) were applied for dereplication, and in silico fragmentation tools combined with classical molecular networking (MN) [40], feature-based molecular networking (FBMN) [41], Dereplicator+ [42], network annotation propagation (NAP) [43], Moldiscovery [44], MS2LDA [45], and MolNetEnhancer [46] resulted in level 2 identification according to the Metabolomic Standard Initiative (MSI) [47]. In order to evaluate the concentration of metabolites in the samples, we applied the FBMN tool using the relative abundance of ions from the data processing performed using MzMine2 software. To assist in statistical analyses, we also used the MS-DIAL and Progenesis QI tools.

The tools employed in this study provided candidates with high confidence in structural annotation and can guide the discovery of biologically active compounds.

The remarkable biotechnological potential of cupuassu pulp is evidenced by the wide molecular diversity of compounds identified.. The evaluation of the metabolic content using computational tools allowed the structural annotation of 20 metabolites with elevated confidence level, in addition to the complexity of substances derived from the triterpenoid oleanane (Table 1). Inspection of the hits suggested by the GNPS spectral library identified spectral library matching candidates with similarity greater than 90%, *m*/*z* error (ppm) less than 10, and the most shared peaks in the MS/MS spectrum. All the annotated metabolites have been previously described in the *Theobroma* genus.

The library matches obtained using the classical molecular network (MN) yielded a total of 1220 hits with 315 unique library compounds. The propagation of structural annotation was performed using in silico fragmentation tools, automated chemical classification, and molecular fragmentation data decomposition tools such as network annotation propagation (NAP), Dereplicator+, Moldiscovery, MS2LDA, and MolNetEnhancer. The Moldiscovery tool yielded a total of 2495 unique metabolites, whereas the in silico dereplication tool Dereplicator+ yielded 341 unique metabolites, and the tool NAP yielded 196 metabolites by NAP-fusion in silico prediction. The MolNetEnhancer tool showed a wide metabolic diversity, mainly polyphenols, organic acids, and terpenes.

### 3.2. Assessing the Effectiveness of Metabolite Extraction from Cupuassu Pulp by PSI-HRMS/MS and LC-HRMS/MS

**Figure 1:**
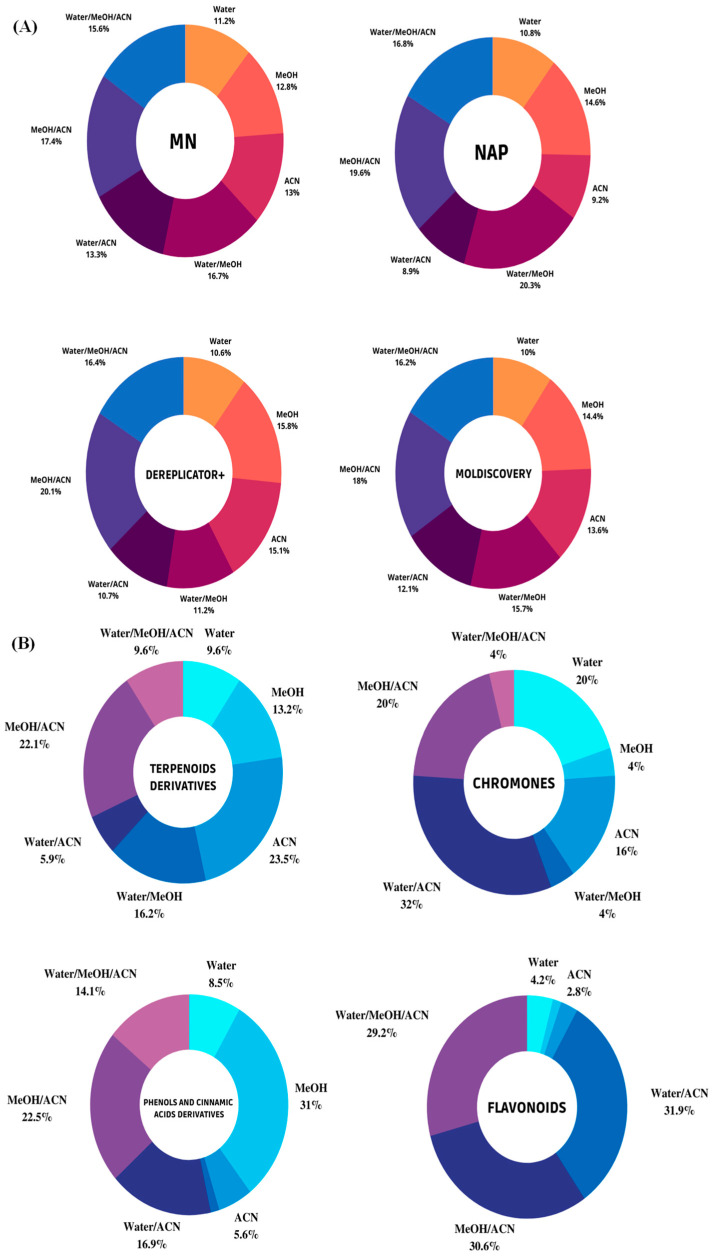
(**A**) Circle diagrams illustrate the percentages of unique metabolites obtained by classical molecular networking (MN), Dereplicator+, Moldiscovery, and NAP fusion from LC-MS spectra of cupuassu (*T. grandiflorum*) pulp extracts. (**B**) Impact of solvent used on the percentage of major chemical classes detected by LC-HRMS/MS analysis in positive ion mode ESI incupuassu pulp extracts.

The efficiency of metabolite extraction from cupuassu pulp using solvents of different polarities and mixtures thereof was evaluated by both, PSI-HRMS/MS and LC-HRMS/MS. Extracts were prepared in duplicate using H_2_O, MeOH, ACN, H_2_O/MeOH (1:1, *v*/*v*), MeOH/ACN (1:1, *v*/*v*), ACN/H_2_O (1:1, *v*/*v*), and H_2_O/MeOH/ACN (1:1:1, *v*/*v*/*v*), respectively. The extraction potential of pure solvents and mixtures thereof can be evaluated according to their physicochemical properties, such as the dielectric constant, i.e., the capacity of a solvent to separate electrolytes into ions [48]. Given that interatomic and intermolecular attractions influence the dielectric constant, mixtures of polar protic solvents (water and methanol) and polar aprotic solvent (CH_3_CN) were used in binary and ternary solvent mixture systems. This parameter was evaluated using the most common mixture analysis method, which employs a weighted average of the mixture components [49]. In order to predict the appropriate composition for the extraction of bioactive metabolites and chemical markers from cupuassu pulp, H_2_O (dielectric constants of 80.00), MeOH (dielectric constants of 33.00), ACN (dielectric constants of 37.50), H_2_O/MeOH 1:1 (dielectric constants of 56.50), MeOH/ACN 1:1 (dielectric constants of 35.25), ACN/H_2_O 1:1 (dielectric constants of 58.75), and H_2_O/MeOH/ACN (dielectric constants of 50.17) [50] were evaluated according to the number of detected hits employing molecular networks, metabolic dereplication tools, and in silico fragmentation tools such as classical molecular networking (MN), network annotation propagation (NAP), Dereplicator+, and Moldiscovery (Figure 1A).

A large number of hits was detected by LC-HRMS/MS-based untargeted metabolomics. However, only the unique metabolites provided by each tool were evaluated. In addition, these tools allowed us to discriminate ions of interest in these datasets and to assess the abundance of compounds in the samples by analyzing the ion area, since all extracts were prepared and analyzed at the same concentration. This information helped predict which extraction system was most effective in extracting the chemical markers and biologically active substances of interest for this research. Qualitative evaluation of the chemical constituents of the sample through analysis of the number of unique metabolites obtained after inspection of the hits generated by the MN, NAP, Dereplicator+, and Moldiscovery tools showed that a greater number of metabolites was putatively annotated in the MeOH/ACN extracts (17.4%), H_2_O/MeOH (16.7%), and H_2_O/MeOH/ACN (15.6%) using molecular networking (MN), indicating a possibly better efficiency of extraction of known metabolites using solvent mixtures rather than pure solvent. Correspondingly, NAP, Dereplicator+, and Moldiscovery yielded a greater number of unique metabolites for extracts prepared with MeOH/ACN (19.6% for NAP, 20.1% for Dereplicator+, and 18% for Moldiscovery) and H_2_O/MeOH/ACN (16.8% for NAP, 16.4% for Dereplicator+, and 16.2% Moldiscovery) (Figure 1A). ACN proved to be a selective solvent in the extraction of medium-polarity compounds, even in combination with polar protic solvents such as water and methanol.

Combining the outputs from molecular networking (MN), network annotation propagation (NAP), and Dereplicator+ with MolNetEnhancer, it was possible to substantially increase the number and diversity of metabolite annotations. At the “class” and “subclass” level, flavonoids, terpenoid derivatives, chromones, and phenol and cinnamic acid derivatives dominated. More than 50% of the putatively annotated metabolites belong to the terpenoid derivative class. Due to its predominantly low-polar characteristic, pure ACN was the most selective solvent in the extraction of these metabolites (23.5%), followed by ACN/MeOH (22.1%), and MeOH/H_2_O (16.2%), with the latter proving particularly effective in extracting glycosylated terpenoids.

Regarding the chromone class, pure water (20%) and mixtures containing ACN, such as H_2_O/ACN (32%) and MeOH/ACN (20%), proved the most effective extractor systems, while methanol was inefficient in the extraction of this class. Phenols and cinnamic acid derivatives are widely occurring metabolites in plants, which were predominantly extracted using methanol (31%) and MeOH/ACN (22.5%), as they are low-molecular-weight polar molecules. On the other hand, MeOH/ACN (30.6%), H_2_O/ACN (31.9%), and H_2_O/MeOH/ACN (29.2%) were more selective than pure solvents in the extraction of flavonoid compounds (Figure 1B).

**Figure 2:**
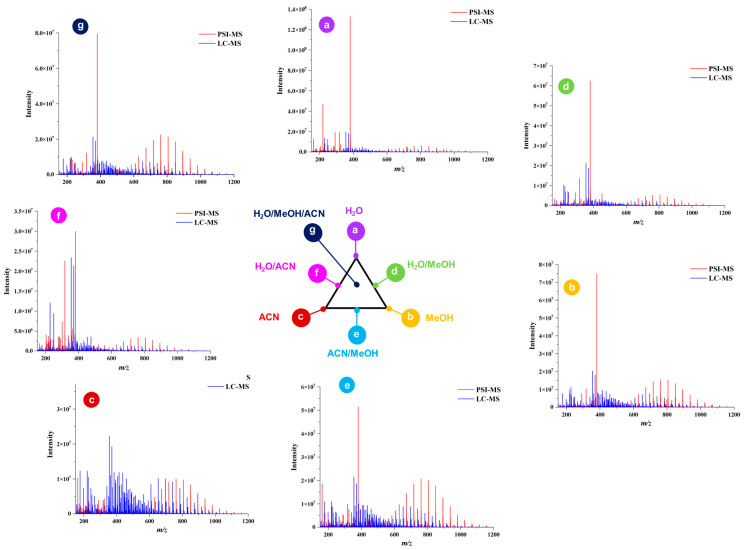
Qualitative comparison of the chemical profiles obtained by paper spray ionization mass spectrometry (PSI-MS) and liquid chromatography–mass spectrometry (LC-MS) from cupuassu (*T. grandiflorum*) pulp extracts prepared with acetonitrile/methanol/water.

Comparison of PSI-HRMS/MS and LC-HRMS/MS in metabolic profiling of cupuassu pulp extracts showed, that LC-HRMS/MS detected more secondary metabolites than PSI-HRMS/MS, reflecting the different physicochemical properties of paper and a C18 stationary phase. A complexity of low-molecular-weight metabolites was detected byLC-HRMS/MS, while PSI-HRMS/MS showed better efficiency in the analysis of terpenoid derivatives, which is the class of compounds mostly found in the samples. In addition, a smaller number of ions was detected by the PSI-HRMS/MS technique regardless of the extractor system used (Figure 2).

**Figure 3:**
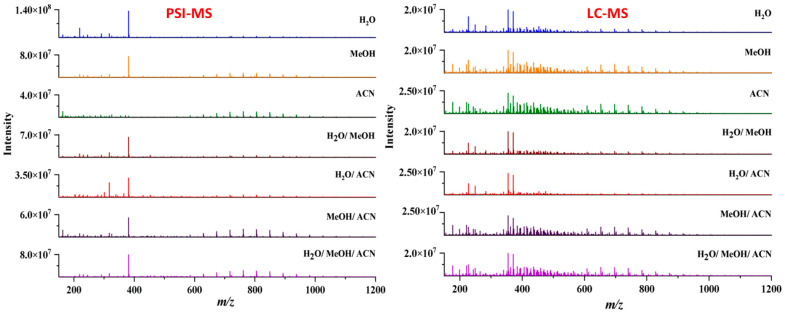
Comparison of chemical profiles obtained by paper spray ionization–mass spectrometry (PSI-MS) and liquid chromatography–mass spectrometry (LC-MS) for the different extraction solvents.

**Figure 4:**
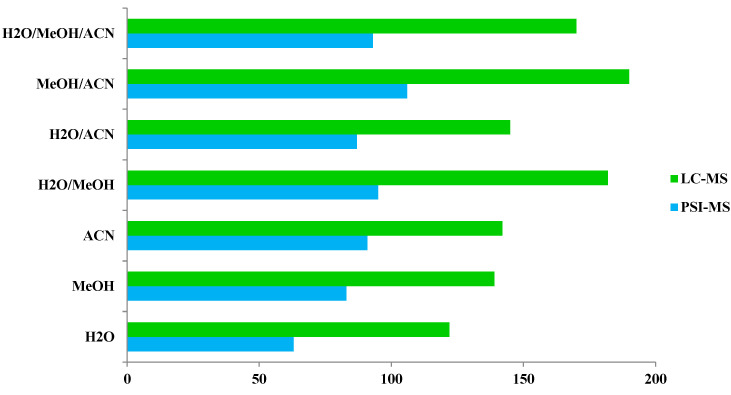
Comparison of the number of compounds detected by paper spray ionization mass spectrometry (PSI-MS) and liquid chromatography–mass spectrometry (LC-MS) in the different cupuassu pulp extracts.

Examination of phytochemical content showed that PSI-MS yielded the largest number of metabolites in MeOH/ACN extracts of cupuassu pulp extracts, while the extractor systems MeOH/H_2_O and MeOH/ACN gave the best results for RPLC-MS (Figure 3 and Figure 4). The paucity of metabolites found in the aqueous extract indicates a low content of highly polar compounds such as sugars and heterosides or failure of their detection by the techniques used. The PSI-MS technique detected fewer phenolic compounds than LC-HRMS/MS irrespectively of the extraction solvent used. (Overall, PSI-MS detected between 60 and 100 compound in the various extracts (Figure 4). LC-MS, in comparison, yielded between 125 and close to 200 metabolites, with water and acetonitrile/methanol being the least and most effective extraction solvent, respectively.

**Figure 5:**
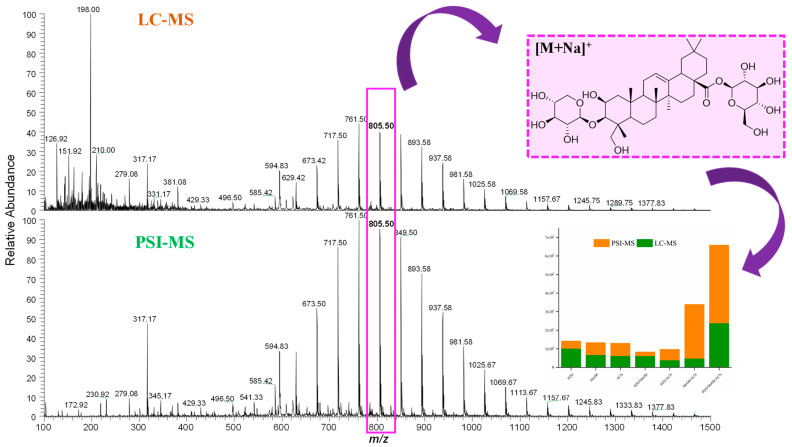
LC-MS and PSI-MS mass spectra showing the extraction efficiency of oleanane triterpenoid derivatives and their abundance according to the extraction system using mixtures of acetonitrile/methanol/water from cupuassu (*T. grandiflorum*) pulp extracts.

After chemical characterization of cupuassu pulp extracts, 22 oleanane triterpenoids derivatives were detected, whose mass difference of 44 Da indicates a variation of C_2_H_4_O in the terpene structure, which is highlighted in the molecular network presented in Figure 5. It was observed that the oleanane triterpenoid derivatives are the major constituents found in these samples. When investigating the influence of PSI-MS and LC-MS techniques on the extraction of oleanane triterpenoids, a greater abundance of this group of metabolites was observed in the PSI-MS spectrum (Figure 5). Considering that the choice of extraction solvents also influences the extraction yield of these compounds, the abundance of the major oleanane triterpenoids derivatives, i.e., the asiatic acid compound of *m*/*z* 805.50 [M+Na]^+^, was evaluated. Based on the physicochemical properties of Asiatic acid, it can be concluded that it is a polar compound due to the presence of hydroxyls and two sugar molecules. Thus, when evaluating the effect of the polarity of the solvent mixture components in the extraction of Asiatic acid, it was observed that the H_2_O/MeOH and H_2_O/ACN extractor systems were unable to completely extract this compound using either of the applied techniques. Based on the area of this compound, the H_2_O/MeOH/ACN system showed greater efficiency in the extraction of Asiatic acid using the PSI-MS technique, while a greater abundance of this metabolite was detected using the LC-MS technique with H_2_O/MeOH/ACN and MeOH/ACN systems (Figure 5).

PSI-MS and LC-MS showed high sensitivity in the detection of the chemical markers of *T. grandiflorum*, the maximum signal intensity of which reached values of 2E8 and 3E7 for PSI-MS and LC-MS for MS1 analysis, respectively.

**Figure 6:**
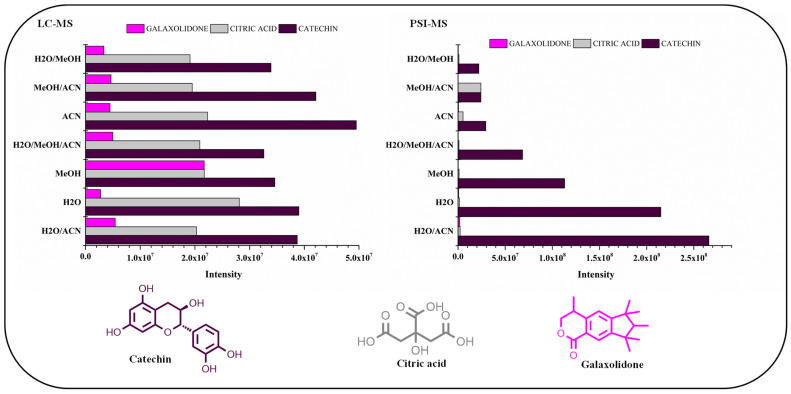
Relative abundance of the major compounds annotated from cupuassu (*T. grandiflorum*) pulp extract samples using mixtures of acetonitrile/methanol/water obtained in LC-HRMS/MS and PSI-HRMS/MS analyses.

Among the tested techniques, PSI-MS proved to be the technique of choice for analysis of this group of compounds, with wide potential for quantification and/or qualitative screening. As a consequence of the structural similarity of the metabolite oleanane triterpenoid derivatives, the influence of the analysis parameters and the properties of the PSI-MS and LC-MS techniques were investigated using abundant metabolites found in samples that have different physicochemical properties such as molecular weight and polarity, including catechin, citric acid, and galaxolidone compounds. The graph presented in Figure 5 was constructed based on the area of ions associated with catechin, citric acid, and galaxolidone compounds, representing the relative abundance of the major compounds annotated from cupuassu pulp extracts samples using mixtures of acetonitrile/methanol/water obtained in LC-HRMS/MS and PSI-HRMS/MS analyses (Figure 6).

The catechin compound was found in high abundance in all extracts and was detected by both techniques. The LC-MS technique was efficient in detecting catechin in all extractor systems used, with the highest abundance found in pure ACN and mixtures with acetonitrile, H_2_O/ACN, and MeOH/ACN. Applying the PSI-MS technique, a greater abundance of catechin was detected in the extracts with H_2_O, H_2_O/ACN, and H_2_O/MeOH, showing that using paper as a substrate influences the extraction and ionization of polar compounds. Previous reports showed that in altering the solvent–substrate systems, variation is observed in the number of detected ions caused by the change in the extraction and ionization efficiency of the analytes deposited on the paper [51]. Our findings show that catechin was easily extracted using polar solvent mixtures, since flavan-3-ols are easily extracted with polar organic solvents and aqueous mixtures [52].

Regarding citric acid and galaxolidone, low detection of these metabolites was observed using the PSI-MS technique. Citric acid was abundantly extracted in all extractor systems and was easily detected by LC-MS. However, PSI-MS was not effective in detecting this metabolite, possibly because its low molecular weight and its highly polar characteristic are factors that can influence the PSI analysis since a chromatographic separation on paper is possible due to the different interactions that occur between the substrate and the analyte. Such effects have been reported before [53]. This on-paper separation may be responsible for the reduction in sensitivity of the analysis of highly polar analytes such as citric acid, as the paper substrate can retain analytes with hydrophilic properties, resulting in low separation [54]. The methanol used for paper spraying also influences analyte extraction, transportation, and spray ionization, as the solvent transports the analytes to the spray tip, extracts the matrix components, and transfers the analytes to the MS system [55]. Concerning the compound galaxolidone, the methanol-extracting solvent was more efficient in extracting this metabolite using the LC-MS technique. However, evaluation of galaxolidone using the PSI-MS technique shows that many factors may have influenced the detection of the low abundance of this analyte using the chromatographic technique. Many factors such as the solubility of the compound in the acidified solvent that transports the metabolite to the spray tip, suppression of ionization by other matrix components, the volatilization capacity of the compound using the ambient ionization method, and the porosity of the paper substrate may have affected the analysis of galaxolidone using PSI-MS. Although the PSI technique was not as efficient as LC-MS in the detection of citric acid and galaxolidone, it was successfully employed in the detection of other biologically active metabolites such as catechin and other flavonoids. In addition, PSI offers many advantages over other chromatographic techniques such as reduced analysis time and solvent consumption.

### 3.3. Determination of Antioxidant Properties Using Molecular Docking

The established antioxidant activities of the major metabolites were explored, and catechin, epigallocatechin-3′-glucuronide, citric acid, 5,7,8-trihydroxyflavanone, and asiatic acid were identified as the main bioactive components; we then undertook a molecular docking study. Two enzymes were used in this docking study: tyrosinase and PRDX5. Tyrosinase is considered the main active enzyme in the biosynthesis of the pigment melanin [32]. Since tyrosinase is known as the limiting-step enzyme in the process of melanogenesis, its connected inhibitors have become very important, considered depigmenting agents in disorders related to hyperpigmentation. Currently available tyrosinase inhibitors are toxic in nature and/or less efficient, and there is a constant requirement for better inhibitors from nature, as they may cause no or less harmful side effects. PRDX5 has antioxidative and cytoprotective functions during oxidative stress. In order to determine the differences in binding, as well as the interactions between the compound structures and the two proteins, rigid receptor docking (RRD) experiments were performed [33].

All docking poses were compared and analyzed relative to quercetin that served as a standard antioxidant. First, a docking experiment was performed on the crystal structure of tyrosinase taken from the protein data bank of *Bacillus megaterium* with identification PDB: 3NM8 [32]. Tyrosinase is the most widely distributed enzyme, with copper as its main constituent, belonging to the class of the type 3 copper enzyme family, and is involved in the formation of melanin pigment in a number of organisms. The second docking experiment was carried out on human peroxiredoxin 5 (PDB code 1HD2), which belongs to the widely expressed six-member peroxiredoxin family of antioxidant enzymes [29]. Peroxiredoxins reduce hydrogen peroxide and alkyl hydroperoxides by reducing equivalents from thiol-containing donor molecules, for example, trypanothione, thioredoxin, glutathione, and AhpF, and they are found in both prokaryotes and eukaryotes. 

**Figure 7:**
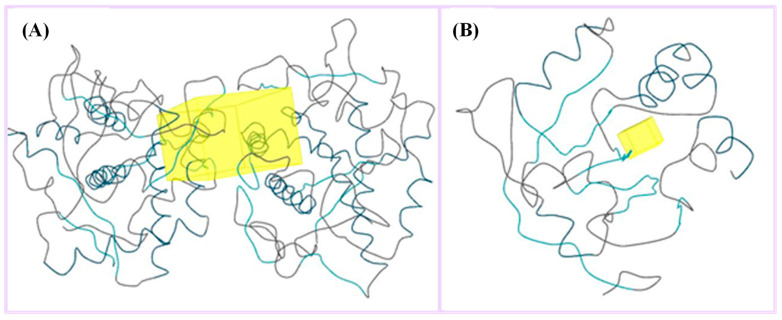
(**A**) Binding site (yellow color) of tyrosinase from *Bacillus megaterium*; (**B**) binding site (yellow color) of human peroxiredoxin 5.

The five major metabolites were subjected to docking experiments, and their interactions with tyrosinase and PRDX5 were investigated in detail, as shown in Figure 7. Based on binding energies and residues interacting with the structures, the best-docked complexes were selected for analysis. Compared to quercetin, only 5,7,8-trihydroxyflavanone yielded a better docking score for tyrosinase. In the case of PRDX5, the docking score of 5,7,8-trihydroxyflavanone (−7.0 kcal/mol) was only surpassed by that of epigallocatechin-3′-glucuronide (−7.7 kcal/mol). Together with asiatic acid (−6.8 kcal/mol), the docking scores of these three compounds were better than that of the standard quercetin (-6.3 kcal/mol) (Table 2). Based on these results, further analyses were conducted with 5,7,8-trihydroxyflavanone. 

**Figure 8:**
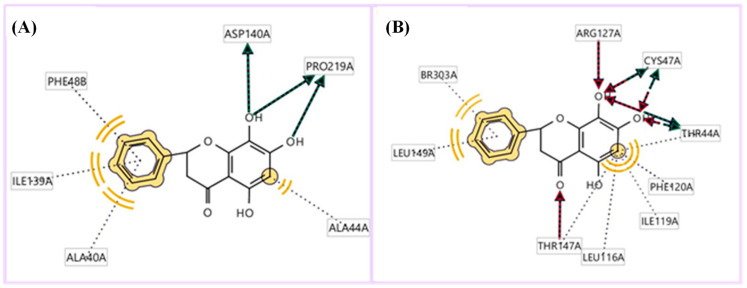
LIGPLOTs showing interacting residues of (**A**) tyrosinase complex and (**B**) human peroxiredoxin 5 with 5,7,8-trihydroxyflavanone. Red arrow dotted lines, HBAs; green arrow dotted lines, HBDs; yellow lines, H interactions.

LIGPLOTs for 5,7,8-trihydroxyflavanone are shown in Figure 8A,B. 5,7,8-trihydroxyflavanone interacts with tyrosinase, forming H interactions involving residues Ala44A, Ala40A, Ile139A, and Phe48B. Tyrosinase residues Asp140A and Pro219A were involved in hydrogen bonding, whereas with Human Peroxiredoxin 5, the 5,7,8-trihydroxyflavanone interacts to form H interactions with Thr44A, Phe120A, Ile119A, Leu116A, Leu149A, and Br303A. Human peroxiredoxin 5 residues Cys47A, Arg127A, and Thr44A were involved in hydrophobic interactions, whereas Cys47A and Thr44A were involved in hydrogen bonding.

### 3.4. Pharmacophore Evaluation

**Figure 9:**
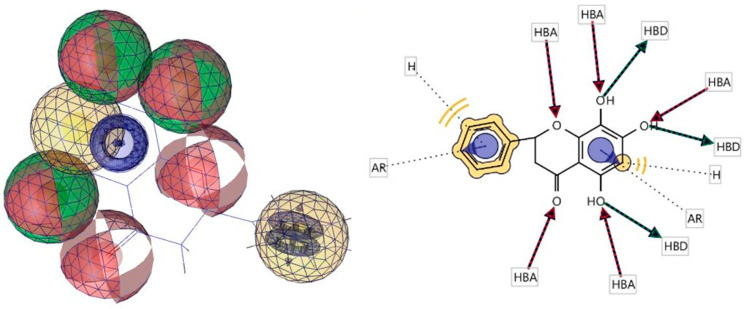
Three-dimensional and two-dimensional representations of pharmacophoric features of 5,7,8-trihydroxyflavanone used in three-dimensional pharmacophore generation. Red, HBAs; green, HBDs; yellow, H; purple, Ar.

Using the lowest-energy conformers of 5,7,8-trihydroxyflavanone, a pharmacophore model was generated by performing a pharmacophore evaluation experiment [31]. The generated model of the pharmacophore has four key features, which are described as hydrogen bond acceptors (HBAs), hydrogen bond donors (HBDs), hydrophobic interactions (H), and aromatic interactions (Ar). Representations of 3D and 2D pharmacophoric features of every compound structure are shown in Figure 9.

## 4. Conclusions

Among the different solvent systems tested for the extraction of metabolites from cupuassu (*T. grandiflorum*) pulp, a 50:50 mixture of methanol and acetonitrile yielded the largest number of metabolites extracted, with LC-MS detecting close to 200 metabolites, whereas PSI-MS detected only a little bit more than 100 compounds. The performance of PSI-MS was found to be influenced by the hydrophilic properties of the analytes, their solubility in acidified methanol, which was used as spray solvent, and their interactions with the paper substrate. Molecular docking analysis of the five major metabolites showed 5,7,8-trihydroxyflavanone to bind particularly well to both, tyrosinase and peroxiredoxin 5, the former catalyzing the oxidation of L-tyrosine to dopaquinone, a precursor of melanin, while the latter is an important cytoprotective enzyme that that inhibits endogenous and exogenous peroxide accumulation. A pharmacophore model of 5,7,8-trihydroxyflavanone revealed a number of molecular features for its recognition by receptors that may guide future studies on the selection and design of new antioxidant structures. 

## Figures and Tables

**Table 1:** Results of the metabolite annotation of cupuassu (*T. grandiflorum*) through LC-HRMS/MS analysis in positive ion mode ESI.

Exact Mass (Da)	Molecular Formula	Error (ppm)	Metabolite Name	Chemical Structure	Chemical Class
290.079	C_15_H_14_O_6_	8	Catechin	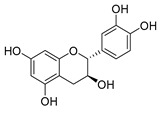	Flavonoid
496.1216	C_22_H_24_O_13_	10	(−)-Epigallocatechin-3′-glucuronide	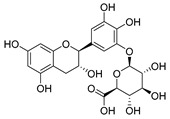	Flavonoid
322.1205	C_20_H_18_O_4_	2	5,7-Dihydroxy-8-prenylflavone	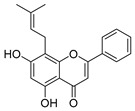	Flavonoid
270.0528	C_15_H_10_O_5_	1	3,5,7-Trihydroxyflavone	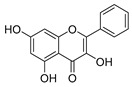	Flavonoid
272.0684	C_15_H_12_O_5_	3	5,7,8-trihydroxyflavanone	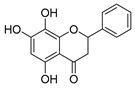	Flavonoid
432.1267	C_18_H_24_O_12_	0	2-Methyl-4-oxo-4H-pyran-3-yl 6-*O*-(4-carboxy-3-hydroxy-3-methylbutanoyl)-beta-D-glucopyranoside	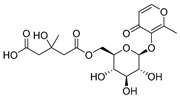	Chromone
260.1048	C_15_H_16_O_4_	10	Heteropeucenin	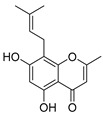	Chromone
192.027	C_6_H_8_O_7_	0	Citric acid	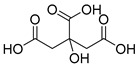	Organic acid
178.0629	C_10_H_10_O_3_	1	4-Methoxycinnamic acid	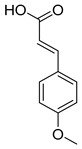	Phenylpropanoid
134.0731	C_9_H_10_O	1	Cinnamic alcohol	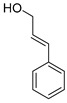	Phenylpropanoid
430.1838	C_20_H_30_O_10_	0	2-Phenylethyl 6-*O*-(6-deoxy-alpha-L-mannopyranosyl)-beta-D-glucopyranoside	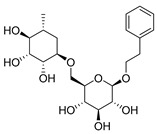	Phenylpropanoid
272.1776	C_18_H_24_O_2_	0	Galaxolidone	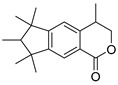	Terpene
250.1568	C_15_H_22_O_3_	9	Hydroxyvalerenic acid	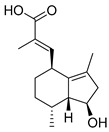	Terpene
274.1568	C_17_H_22_O_3_	10	Nimbidiol	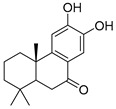	Terpene
332.1987	C_20_H_28_O_4_	9	7|A,15-Dihydroxydehydroabietic acid	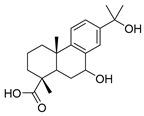	Terpene
250.1568	C_15_H_22_O_3_	10	2-[(2S,4aR,8aS)-2-hydroxy-4a-methyl-8-methylidene-3,4,5,6,7,8a-hexahydro-1H-naphthalen-2-yl]prop-2-enoic acid	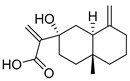	Terpene
320.2351	C_20_H_32_O_3_	10	(1R,4aR,5S)-5-(3-hydroxy-3-methylpent-4-enyl)-1,4a-dimethyl-6-methylidene-3,4,5,7,8,8a-hexahydro-2H-naphthalene-1-carboxylic acid	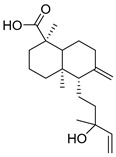	Terpene
442.381	C_30_H_50_O_2_	0	Allobetulinol	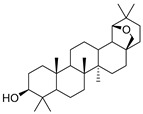	Terpene
488.3501	C_30_H_48_O_5_	4	Asiatic acid	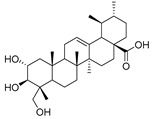	Terpene
426.3861	C_30_H_50_O	2	Lup-20(29)-en-3-ol	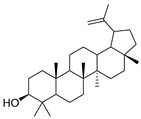	Terpene

**Table 2:** Docking analysis of the five major metabolites from cupuassu (*T. grandiflorum*) on different protein receptors with respect to quercetin.

Compound	Docking Score (kcal/mol) PDB:3nm8 (Bacterial tyrosinase)	Docking Score (kcal/mol) PDB: 1HD2 (Human Peroxiredoxin 5)
Quercetin (standard)	−10.4	−6.3
Citric acid	−5.8	−4.5
Catechin	−7.5	−6.1
5,7,8-Trihydroxyflavanone	−11.4	−7.0
Asiatic acid	−8.0	−6.8
Epigallocatechin-3′-glucuronide	−9.0	−7.7

## Data Availability

All support data used in this study are available from the authors. Data is not publicly available due to privacy.
